# Social Support in Two Cultures: Everyday Transactions in the U.S. and Empathic Assurance in Japan

**DOI:** 10.1371/journal.pone.0127737

**Published:** 2015-06-24

**Authors:** Beth Morling, Yukiko Uchida, Sandra Frentrup

**Affiliations:** 1 Department of Psychological and Brain Sciences, University of Delaware, Newark, DE, United States of America; 2 Kokoro Research Center, Kyoto University, Kyoto, Japan; The University of Chicago, UNITED STATES

## Abstract

We studied received social support using the cross-cultural method of situation sampling. College students from the US and Japan described and rated recent examples of received social support, both everyday support as well as support in response to stress. Middle class, European-American (EuA) students’ situations fit a model in which support is frequent and offered freely in interactions, even for relatively minor issues. Even when it’s unrequested, EuA support makes recipients feel in control, and support-givers are perceived to have acted by free choice. In contrast, results suggest that middle-class Japanese (Jpn) contexts favor support that is empathic and responsive to the recipients’ degree of need. Japanese support was experienced positively when it was emotional support, when it was in more serious situations and when the support was rated as needed by the recipient. In Japan, although problem-based support is most common, it is not particularly positive, apparently because it is less likely to be perceived as needed.

## Introduction

“One time when I was very stressed and negative…I had a research paper due in a week or so and was panicking. Anyway, I called my friend and he talked to me. I just vented and he listened and gave me some advice and a new perspective.” (Situation from the US)

“I did not go to my economics class one day. My friend took notes for me. I was happy that I have good friends.” (Situation from the US)

“After class, one of my classmates called to stop me to tell me I was about to leave my umbrella in the classroom.” (Situation from Japan)

“Recently I’ve been feeling a bit gloomy and unable to pull myself out of it, but I have been acting like everything is okay…one of my friends who sensitively realized something was wrong, worriedly asked me “Are you alright?” very nonchalantly. I gained so much relief knowing that I had someone who was looking after me.” (Situation from Japan)

Social support is a key type of human social interaction. People participate in helpful interactions regularly—even daily[1]. Applied research on social support documents the link between perceived support and health outcomes during stress[2–5]. But as the opening examples suggest, people receive assistance not only in stressful situations but also in everyday events (see also Tables 1 and 2).

**Table 1:** Examples of Emotional Social Support from the United States and Japan.

	Japan		United States	
	Requested	Not requested	Requested	Not requested
N of situations listed:	6	20	25	67
Average time ago (days):	9.67	20.11	27.85	29.33
% of situations provided by friends and family	100%	81.2%	85.7%	98.3%
Examples of Emotional support	When I said I wanted to eat Korean food out, my friend from class went with me even though they had another small commitment. Even after that we went out (which was selfish on my part) and it was a blast.	I wrote a journal entry on my Facebook (one where I wasn’t feeling well, and was depressed, though I didn’t outright express it in the entry), and many of my friends commented by saying “Come over to my house again!	Last week, I was feeling especially stressed out with my classes and completely homesick. . . .Two days before the weekend, my dad said that he would come down to visit…He took the time out of his busy schedule to come visit me and to see my singing group perform…he said he wanted to…be there for me…	The cat I have had for 11 years and had grown to be close with died. My girlfriend supported me by talking me through it every day until it was better. I initially felt terrible, but after talking I felt much better
	I was so busy once I became exhausted, and when it got to the point where I didn’t know what I should do, I called my parents and talked on and on about my problems. I calmed down and was able to turn my feelings around. Firmly listening to what I had to say as well as talking about my good points cleared up my anxiety and made me feel at ease.	My friend who realized I was feeling down took the time to talk to me and give me some advice. She said “Let’s forget our troubles” and spent several hours hanging out with me in the shopping district of the city. In the end I felt so much better. Even though I never said anything about feeling down, the fact that my friend is kind enough to realize it made me so happy.	I was unsure of which major to pick, so I asked my dad for help…He encouraged me and allowed me to choose whichever major I wanted. It made me feel more confident to decide, knowing I had his support.	When I was sick last week, my girlfriend came to see me from her school in the city. . . .She. .stood by my side and gave me support because being sick away from home is not easy and she understood that. She did not pressure me to go out and do anything. She just remained at my side. It made me feel very happy and loved, especially because I did not even ask for the support.

**Table 2:** Examples of Problem-based Social Support from the United States and Japan.

	Japan		United States	
	Requested	Not requested	Requested	Not requested
N of situations listed:	87	100	83	53
Average time ago (days):	14.49	12.59	15.22	17.25
% of situations provided by friends or family	72.4%	65.0%	84.0%	78.8%
Examples of problem-based support	One time, when going to hang out with our friends, my friend kindly let me ride on the back of her bike when I asked her, since I don’t have a bike.	I was trying to get my bicycle from the rack. The bike next to mine was strangely caught on mine, and when it looked like I would not be able to move it at all, my friend who was with me helped me out. I have a very close relationship with this friend	My mom helped me when I was at work. I worked really late and my mom woke up and came to get me from the mall because I didn’t have my car. She had to wake up and come drive to me. I really appreciate her for being there whenever I need her.	I needed a ride home for the weekend and could not find one. My uncle called me up and said he would drive down, get some dinner with me, and drive back up. This was really out of his way and I really appreciated him using his resources for me.
	I was the manager of a mixer, but I was at the point where I couldn’t do the seating arrangements or the reservations. When I asked friend T, whom I met by chance in the cafeteria, he graciously offered to make the reservations, take care of the seating arrangements, and do the planning. As a result the mixer was a success. I felt both sorry and hugely grateful for the help.	When I left my friend’s house after being there very late, my friend took me home because he said it was dangerous for a girl to go home alone. I was so happy he did that for me.	I asked my friend to come with me on a double date. The girl I was going to go out with had a friend and needed someone to go with her. For the most part, my friend did not say anything bad, except when he almost told her about another girl that I was seeing. During the date, I was fairly relaxed but tensed up when my friend began talking about this other girl.	I got in a big argument with someone a few weeks ago. It was a stupid fight but we were yelling at each other. Without even asking my best friend stepped in and defended me. It made me really happy. She had my back no matter what and helped me win the argument.
	Since I didn’t know the computer operations for my information technology class, I asked my friend who was sitting next to me. Upon doing so, my friend taught me the operating methods and I was able to solve the problem. I felt grateful for the help. Around me there were other students, a number of TAs and the teacher.	When I was conducting an experiment in the research lab, I was using a machine for the first time and didn’t exactly know how to operate it. I tried … until finally a grad student showed me how. I was thinking I would steadily have gotten it through trial and error, so his help was a bit appreciated but also a bit of a hindrance.	In a class where I get 2 possible rewrites to try for an A, I received a B on my paper. I approached my professor as to what I needed to do to get an A and he took 10 minutes after class to show me what I could do differently. I rewrote it and got an A. .	I recently decided to send a school transfer application out and my mom and dad have been particularly supportive of my choice. They’ve helped me decide where [to apply]. . They just wanted me to be happy and sat down with me on several occasions to look over choices and think of what they could do to make my decision less difficult.

In the present article, we explore how two sociocultural contexts (those of middle class college students in the United States and Japan) shape the meaning of social support. According to several recent studies comparing middle class East Asian and European-American contexts, social support is culturally shaped. Specifically, in middle class European-American cultural contexts, social support is transacted between autonomous people, in which one person offers support and the other receives it, often via request[6]. In the cultural model of middle class Japanese cultural contexts, social support takes place in a network of empathy or emotional support[7–9]. In such a network, people may benefit from receiving support mainly when others have detected their needs.

The present research uses the situation sampling methodology (a core methodology in cultural social psychology[10,11],to test these two models of support in everyday contexts. Our examples include support in response to stress, as well as more mundane helpfulness. We collected naturalistic examples of received social support including both emotional support (i.e., reassuring others of one’s love and care) and problem-based support (i.e., information, advice, or tangible help).

### Middle Class American Social Support: Common Transactions Support Autonomy

In European-American, middle-class contexts it is common and effective to request support from others during stress, compared to East Asian contexts[12–14]. Everyday middle class European-American contexts are pervaded with social support: in one study, 35% of social interactions involved it (twice as often as in Japan)[7].

Not only is support more common in middle-class European-American (EuA) settings, it is also enacted in line with particular EuA values. Kim, Taylor, Sherman, and colleagues[6] show that in such settings, the experience of social support takes the form of a transaction between autonomous individuals. A transaction model resonates with the independent model of the self, which is conveyed in dominant EuA cultural practices, discourses, and products[15,16] and supports a view of the person as autonomous, positive, and in control. Indeed, in European-American relationships, neither party in a friendship may be obligated by duty; rather, both are seen as autonomous participants in a voluntary relationship [17–19]. Others are free to accept or deny a request for support, and receivers can construe another’s offer of support as a free choice. Middle-class European-American support also affirms individuals. The relationship between perceived social support and well-being was mediated by self-esteem in EuA samples[8,12]. In addition, when EuA college students offered social support to their friends, their goal was to increase their friends’ self-esteem[20].

Receiving social support might produce ambivalent feelings in any culture. In North America, the ambivalence may stem from the benefits of support and the costs of social support to the individual’s sense of competence[21,22]. On the one hand, support benefits people practically and socially. Research shows that social support that is rated as understanding and caring is beneficial in North America[23]. On the other hand, receiving support can make the recipient feel incompetent or helpless[24,25]. European-American cultural models of support might help individuals navigate and resolve some of the ambivalence. In European-American cultural settings, the transaction model might mitigate threats to autonomy: when support is perceived as repayable or has been exchanged equally (presumably, exchanged between autonomous individuals), North Americans are less bothered by it[24,26].

In the present study of social support, we predicted, based on this transactional cultural model in EuA contexts, that when social support is requested by one person from another, it would be rated by the recipient as more beneficial than unrequested support. We also expected European-American social support, overall, to be perceived as freely offered by others and eventually repayable, according to the EuA cultural model of social interactions between autonomous individuals.

### East Asian Social Support: Helpful When it is Truly Needed

In middle class Asian and Asian-American contexts, social support is not only less prevalent[20] but also enacted differently. East Asian contexts support a model of the self as socially connected and interdependent[27], a model that foregrounds accommodation and tuning in to others. In an interdependent cultural context, social support may be modeled on empathy and assurance: People anticipate others’ needs and feel obligated to respond to each other. In such contexts, social support may result in a different kind of ambivalence. On the one hand, people in interdependent cultural contexts feel close when they have their needs met by others: Japanese students offered social support to their friends with the goal of fostering closeness (not self-esteem, as was the case for North Americans) [8,20]. And *amae* scripts can evoke positive feelings of being needed and valued[28,29]. On the other hand, in Asian contexts, people want to avoid burdening others with support requests [1,13,30–34]. As a compromise, experiencing simple emotional connectedness as a form of social support might confer the benefits of social support without the costs of burdening others[31,35,36].

Because of the cultural model of empathy and assurance in Japan, we expected that emotional support, more than problem-based support, would be perceived as beneficial in the everyday support settings we studied. In North America, too, social support that is understanding and caring is beneficial [23]. However, we propose that in Japanese contexts, it will be even more important that offers of help are sensitive to the recipient’s needs, intentions, and wishes[37,38]. A model of the self as embedded, flexible, and sensitive to others is common to many East Asian cultural models of relationship[39,40] and interdependence seems to make advice, such as parenting advice, more effective in Asian-American contexts[41]. Therefore, sensitive support fulfills Japanese cultural values and follows Japanese scripts for dependence and responsiveness[29]. Therefore, in such contexts, support that is offered in serious situations, that is offered when the recipient most needs it, or that is simply emotionally understanding, should be more effective.

In addition, because past research has shown that in stressful situations, *requested* support was not usually helpful for Asian samples because it imposes burdens on others[1,30], we predicted that requested support would be rated as less beneficial than unrequested support.

### Goals of the Present Research

Our study capitalized on the features of situation sampling methodology to test these cultural models of received social support. As described in the next section, situation sampling data can quantify the meanings people give to naturally-occurring experiences.

Our research differs from recent cross-cultural social support research that begins with a stressful event and asks about social support in response to it. In this study, we simply asked people for examples of support they had received recently. This method enables us to study social support as an everyday social interaction, rather than only as a response to a particular stressful event.

### Situation Sampling Method

The situation sampling method follows two stages to collect and characterize situations from different cultural contexts[10,42]. In Stage 1, samples of participants in two cultural settings remember and describe recent, actual situations which fit particular criteria. (In the present research, we collected social support situations.) In Stage 2, researchers present a random sample of situations from Stage 1 to new participants from each culture, who rate them on dimensions of interest. Situation sampling allows us to quantify how the situations that surround people every day might be culturally different.

Participants may select, describe, and rate situations in ways that reflect the dominant cultural discourse and that do not threaten culturally valued self-ways. For example, European-Americans may emphasize their own control in a situation or others’ autonomy in providing support, and Japanese may emphasize how others have met their needs.

A strength of situation sampling is that it documents both the qualities of *people* who rate situations, as well as the *situations* that a culture provides. It provides three types of data. First, when Stage 1 participants who write the situations (*authors)* rate their own situations, they might report having experienced their own situations differently (for example, middle-class European-Americans might report feeling more competent and in control than middle-class Japanese). Second, Stage 2 *raters* might respond to situations differently (for example, EuA raters might report higher ratings of competence and control than Japanese raters overall, regardless of the situations they are rating). These first two responses can support hypotheses about culturally-shaped psyches.

Third, EuA and middle-class Japanese (Jpn) *situations* might be rated differently in Stage 2 (for example, EuA situations might be rated as more competent than Jpn situations regardless of who is rating them). Such situation-level differences indicate the affordances of each culture’s situations—how would it feel to encounter the situations in each culture? In addition, whereas the cultural differences in the responses of authors and raters might be confounded by cultural response biases such as modesty or response extremity, Stage 2 raters do not know the situations come from different cultures, so any cultural response biases would be applied to all situations.

### Research Hypotheses

For US social support, past research[20] suggests that EuAs easily transact social support; they offer it freely and feel comfortable requesting it from others. EuA support is likely to reflect an emphasis on individual choice and control. For Jpn social support, past research suggests that effective support is enacted in a close, empathic relationship, but one with support obligations.

First, we made predictions about the two key independent variables—support request (requested or not) and support type (emotional vs. problem-based).

H1) Support *request* will interact with culture, such that EuA requested support will be experienced as more effective than unrequested support[12,31]; whereas in Jpn contexts, requested support will be experienced as less effective[1]

H2) Support *type* will interact with culture, such that for Japanese raters and in Japanese situations, emotional support will be rated more positively (i.e., lower in stress, more competent, more positive emotions) than problem-based support. Emotional support best matches the Japanese emphasis on empathy and assurance in close relationships.

Support type and support request might interact, too. A methodological strength of the current work is that we separated asked- and unasked-for support from problem-based and emotional support. Some research has compared “explicitly requested” to unrequested support, but did not distinguish problem-based from emotional support[13,31]. Other research has compared emotional to other forms of support, but did not distinguish requested from unrequested support[20,35]. Because past research has not tested how support request and support type interact, we took an exploratory stance when testing these interactions.

Next, our analysis led to the following prediction about the importance of needed support in Japan:

H3) Especially in Japan, support should be rated more effective when it is reported as *needed* by the support recipient. If empathy, sympathy, or interdependence do matter more in Japan[38,41] then Japanese support should be experienced more favorably when it is attuned to the recipient’s needs. Therefore, the more a situation author conveys that support was needed, the more positively that situation may be rated. In addition, situation severity may be another indicator of how much support is needed. In more stressful situations, Japanese support recipients should rate the support especially favorably.

Next, our analysis of the transaction model of support in EuA led us to make this prediction:

H4) By EuA raters, and in EuA situations, the support-provider is more likely to be portrayed as acting out of free choice than by Jpn raters and in Jpn situations. The support recipient is more likely to see the support as repayable.

Finally, while not central, the following predictions constitute conceptual replications of past cultural work:

H5) By EuA raters, and in EuA support situations, the support recipient will feel more competent and in control than by Jpn raters and in Jpn situations.

H6) Jpn raters and Jpn situations will be rated higher in the support recipient having burdened others than EuA raters and situations.

## Method

### Ethics Statement

We report how we determined our sample size, all data exclusions, all manipulations, and all measures in the study. The project was reviewed and declared as Exempt by the University of Delaware Institutional Review Board.

### Overview

In Stage 1, we collected examples of received social support from college students in the United States and Japan. Each participant in Stage 1 also rated how each situation felt, on positive and negative emotions, autonomy, control, and relatedness.

In Stage 2, we selected a random sample of Stage 1 situations from each culture, translated them, and presented them on a questionnaire to a new sample of Jpn and EuA college students. Stage 2 students rated each situation on feelings of stress, burden, competence, the support-giver’s free choice, and the ability to repay. Importantly, Stage 2 respondents did not know that the situations had come from different cultures, enabling us to compare situation country as a within-subjects variable.

### Stage 1

#### Participants

To collect situations in Stage 1, we planned for and recruited 52 students (27 female) from the University of Delaware in the US (*M* age = 18.40; *SD* = 0.69) and 52 students from Kyoto University (26 female) in Japan (*M* age = 19.40; *SD* = 0.93). University of Delaware students earned course credit for participation; Kyoto University students earned 1000 yen (about $12.00). We excluded data of 2 American students who were born abroad.

#### Materials and procedure

Participants worked individually in group sessions. In the first part of the session, they were given the following instructions:

Please think about recent situations in which another person did something for you. You may have asked the person for support, or you might not have asked them for support. You might have been happy with the support, or you might not have been happy with the support. When you think of situations like this, write each one down in the box on one of the pages provided. Write about each one separately on its own page.

Student participants then proceeded to list as many situations as they could in a 20 minute time period, writing each situation on a separate page after the prompt: “A recent situation in which another person did something for me was:” On average, students listed 4.50 (Japan) and 4.44 (US) social support situations.

After listing situations, participants were instructed to return to each situation one at a time. They reported how long ago the situation occurred and identified the person who offered support (for example, “friend,” “brother,” “classmate”). They rated how much they **needed** the support (1 = “did not need at all” 5 = “needed very much”), and indicated whether they **requested the** support in the situation (“Yes” or “No”).

Participants rated the degree to which they felt each of 16 **emotions**, (from 0, “not at all” to 7, “very strongly”). Later, factor analyses indicated that we could combine individual emotions into a positive emotion subscale (the mean of happy, proud, calm, competent, relaxed, elated, and high in self-esteem) and a negative emotion subscale (the mean of angry, indebted, anxious, ashamed, guilty, tense, discouraged, sad, and miserable).

Finally, participants rated three items based conceptually on **control, relatedness,** and **autonomy[43]** with wording adapted from Morling, et al.[10]. We asked, “When you received that support, to what extent did you feel efficacious, powerful, and competent?” (anchored by -3 = *powerless*, *incompetent*, *not efficacious* and +3 = *powerful*, *competent*, *efficacious*; “When you received that support, did you feel connected to or interdependent with the person who provided the support to you? Or, did you feel separate from or independent of them?” (anchored by -3 = *independent*, *separate from the person* and +3 = *interdependent*, *connected to the person*; and “When you received that support, did you feel coerced or forced to accept the support, or did you internally feel like you gave assent to the support, and that you were autonomous and willing?” (anchored by -3 = *coerced*, *forced to do or accept something* to +3 = *autonomous*, *willing*, *assenting*).

#### Coding

Research assistants coded each situation for the type of support received. Two EuAs coded US situations in English and three Jpn coded Japanese situations in Japanese. Each situation was originally coded for one or more of the three categories of support: emotional, instrumental, or informational. As defined by Cohen and Wills[44], *emotional support* is knowing that one is loved and cared for. *Instrumental support* is receiving tangible goods or financial resources. *Informational support* is receiving advice or guidance and “help in defining, understanding, and coping with problematic events” (p. 313). Later, to simplify our dataset and make it comparable to other work[20], we combined the instrumental and informational support categories into a single category called Problem-based support[20]. On a subset of situations, American coders trained to 84% agreement and Japanese coders trained to 93%. Thereafter, all data were coded by both coders and any coding disagreements were resolved through discussion.

Coders rated the **severity** of each situation before help was offered (0 = *not at all severe* to 3 = *very severe*) and rated the **positivity** of the situation after receiving the support (-3 = *very negative* to +3 = *very positive*). American coders’ ratings correlated .79 for severity and .68 for positivity. Japanese coders’ ratings correlated .84 for severity and .86 for positivity. Because of high agreement, we averaged the ratings of the two coders.

### Sampling from Stage 1

To create the questionnaire for Stage 2 we randomly selected up to 20 situations from each cell of a 2 (situation country) x 3 (support type) x 2 (support request) design. As noted, we later combined informational and instrumental support situations into “problem-based” support, as past research has[20]. Past research has not found gender differences so we did not sample on this variable. Tables 1 and 2 present examples of each type of support. To avoid making the questionnaire too long, we divided this set into 4 questionnaires of about 60 situations each. We created a reverse ordered version of each questionnaire, resulting in eight versions total.

Although we attempted to assign 20 situations of each type to each set, there were only 6 Japanese “requested emotional support” situations (obtained from six different authors) and 19 US informational support situations, so we used the full samples of these. Selected Jpn situations were translated into English by an American graduate student fluent in Japanese; these were later checked by a bilingual graduate student. Selected EuA situations were translated into Japanese by a bilingual graduate student and checked by a bilingual faculty member. Culturally specific descriptions (i.e., fraternity or sorority in the US) were replaced by more general descriptions (i.e., club activity).

### Stage 2

#### Participants

We planned 120 and recruited 140 University of Delaware students (68 female) who had identified as “white” in pretesting. We planned 100 and recruited and 97 Kyoto University students (46 female, all Japanese). Age was not collected, but all students were within the traditional undergraduate age of 18 to 23. We eliminated 4 American participants for finishing too quickly and 3 who were raised outside the United States. American participants received course credit; Japanese participants received 1000 yen (about $12).

### Materials and Procedure

We printed each situation at the top of a page followed by six questions. To ask a broader range of questions, we chose to ask different dependent variable questions at Stage 2 than Stage 1. Participants were asked to imagine that they had received the support described, and to rate how they would feel. The dependent variables were developed concurrently in Japanese and English. The first situation on the first page was followed by a long version of the questions that fully explained the rating scales. Every situation thereafter had a shortened version of the questions, as follows.

The question for **stress** read, “To what extent would you feel stress in this situation? Indicate the extent of your feelings of stress on the scale below. If this situation would not affect your feelings of stress, circle N/A below.” The scale anchors were -3 = *I would feel calm and relaxed* to 0 = *N/A* to +3 = *I would feel very stressed*.

Response anchors for feelings of **burden** ranged from 0 = *I would feel not at all obligated*, *burdened*, *or troubling others* to 4 = *I would feel very much obligated*, *burdened*, *troubling others*.

Two scales asked about the **support-provider’s free choice**. One set of response anchors ranged from 0 = *Person giving support did not act out of obligation* to 4 = *Person giving support acted very much out of obligation*. The other set ranged from 0 = *Person giving support did not act out of personal choice* to 4 = *Person giving support acted very much out of personal choice*. We subtracted obligation ratings from personal choice ratings; high scores on this composite indicate that people thought the person giving support acted more out of personal choice.

Response anchors for the opportunity to **repay** the support ranged from 0 = *There would be no opportunity to reciprocate/repay* to 4 = *There would be certain opportunity to reciprocate/repay*.

Finally, response anchors for the **competence** ratings ranged from -3 = *I would feel incompetent/inefficacious* to +3 = *I would feel competent/efficacious*.

## Results

Tables 1 and 2 show frequency, recency, and examples of situations collected in Stage 1. In general, the support happened within the last month and was provided by friends.

### Problem-Based Support is Less Serious

Although not predicted, we noticed that the problem-based support situations were not only more numerous; they also tended to describe everyday events which were less serious (such as missing class, planning an event, or doing homework); in contrast, emotional support situations were in response to a stressor (see Tables 1 and 2). Results support this impression: Problem-based support situations were coded as significantly less severe (*M* = 0.81) than emotional support situations (*M* = 1.23) (γ_20_) = 0.36 (0.11), p < .001 in both cultures (i.e., the interaction with author country was n.s.). Fitting with its reduced severity, problem based-support also happened marginally more recently—fewer days ago, (γ_10_) = 6.97 (4.06), *p* = .089. In sum, problem based situations are more frequent and describe common, minor assistance; emotional support situations are less frequent and describe more serious events.

### Analytic Strategy

Situations are nested within person, so we tested most hypotheses using hierarchical modeling software (HLM7beta[45]). Situations were Level 1; the main Level 2 variable was Country. We tested all 5 dependent variables at Stage 1 (positive emotions, negative emotions, rated autonomy, competence, and relatedness); and all 5 at Stage 2 (rated feelings of stress, feelings of burden, ability to repay, feelings of competence, and relative degree of choice of the support-provider). In Stage 1 data, the number of Level 1 and Level 2 cases was 444 and 104, respectively. In Stage 2 data, the number of Level 1 and Level 2 cases was 12,765 and 230, respectively. Data files (SPSS format) are provided in supplementary files (S1 Datasets for Stage 1 and Stage 2 data).

#### Stage 1 model

Predictors for main effects at Level 1 (Situation Request—no [= 0] or yes [= 1], and Situation Type—problem based [= 0] or emotional [= 1]) were person-mean centered for each author (i.e., creating within-person mean-deviated scores). Interactions were computed from the product of these person-centered main effect predictors. The focal Level 2 independent variable was author country (US = 0, Japan = 1). We also included the person-mean proportions for Situation Request and Situation Type (both grand-mean centered) into Level 2 to control for each author’s average level of asking and average tendency to write each situation type. The general Stage 1 model was:

$$\begin{array}{l} DV=\beta_{{}_{0j}}+\beta_{{}_{1j}}\;\left(situation\;request\right)+\beta_{{}_{2j}}\;\left(situation\;type\right)+\beta_{{}_{3j}}\;\left(situation\;type\;X\;situation\;requested\right)+r_{ij}\\ \beta_{{}_{0j}}=\gamma_{{}_{00}}+\gamma_{{}_{01}}\;\left(author\;country\right)+\gamma_{{}_{02}}\;\left(mean\;situation\;request\right)+\gamma_{{}_{03}}\;\left(mean\;situation\;type\right)\;u_{{}_{0j}}\\ \beta_{{}_{1j}}=\gamma_{{}_{10}}+\gamma_{{}_{11}}\;\left(author\;country\right)+u_{1j}\\ \beta_{{}_{2j}}=\gamma_{{}_{20}}+\gamma_{{}_{21}}\;\left(author\;country\right)+u_{{}_{2j}}\\ \beta_{{}_{3j}}=\gamma_{{}_{30}}+\gamma_{{}_{31}}\;\left(author\;country\right)+u_{{}_{3j}} \end{array}$$

#### Stage 2 model

In Stage 2, predictors were situation request, situation type, and situation country (dummy coded) as well as interactions. The focal Level 2 variable was rater country. Because each respondent was exposed to equal proportions of each situation type, it was not necessary to center Level 1 predictors within-person or re-introduce the person-means in Level 2. The general Stage 2 model was:

$$\begin{array}{l} DV=\beta_{{}_{0j}}+\beta_{{}_{1j}}\;\left(situation\;country\right)+\beta_{{}_{2j}}\;\left(situation\;request\right)\\ +\beta_{{}_{3j}}\;\left(situation\;type\right)+\beta_{{}_{4j}}\;\left(situation\;country\;*\;situation\;request\right)\\ +\beta_{{}_{5j}}\;\left(situation\;country\;*\;situation\;type\right)+\beta_{{}_{6j}}\;\left(situation\;request\;*\;situation\;type\right)\\ +\beta_{{}_{7j}}\;\left(situation\;country\;*\;situation\;request\;*\;situation\;type\right)+r_{{}_{ij}}\\ \beta_{{}_{0j}}=\gamma_{{}_{00}}+\gamma_{{}_{01}}\;\left(rater\;country\right)+u_{{}_{0j}}\\ \beta_{{}_{1j}}=\gamma_{{}_{10}}+\gamma_{{}_{11}}\;\left(rater\;country\right)+u_{{}_{1j}}\\ \beta_{{}_{2j}}=\gamma_{{}_{20}}+\gamma_{{}_{21}}\;\left(rater\;country\right)+u_{{}_{2j}}\\ \beta_{{}_{3j}}=\gamma_{{}_{30}}+\gamma_{{}_{31}}\;\left(rater\;country\right)+u_{{}_{3j}}\\ \beta_{{}_{4j}}=\gamma_{{}_{40}}+\gamma_{{}_{41}}\;\left(rater\;country\right)+u_{{}_{4j}}\\ \beta_{{}_{5j}}=\gamma_{{}_{50}}+\gamma_{{}_{51}}\;\left(rater\;country\right)+u_{{}_{5j}}\\ \beta_{{}_{6j}}=\gamma_{{}_{60}}+\gamma_{{}_{61}}\;\left(rater\;country\right)+u_{{}_{6j}}\\ \beta_{7j}=\gamma_{{}_{70}}+\gamma_{{}_{71}}\;\left(rater\;country\right)+u_{{}_{7j}} \end{array}$$

In both Stage 1 and Stage 2, we conservatively tested the full model with all main effects and interactions (in part because we were exploring interactions between support request and support type). If the higher level, 3-way interaction between rater country, situation type, and situation request was not significant, we eliminated the 3-way interaction and tested a simpler model with only rater country X situation type and rater country X request two-way interactions (entered simultaneously). We estimated simple slopes using an online calculator as needed[46].

In preliminary analyses, we included rater gender in analyses, but found no meaningful or consistent patterns. Age was not collected from participants; in any case, the restricted range on age in this college student sample precludes strong tests of any age-related hypotheses.

### Results and Discussion of Hypotheses

**Fig 1:**
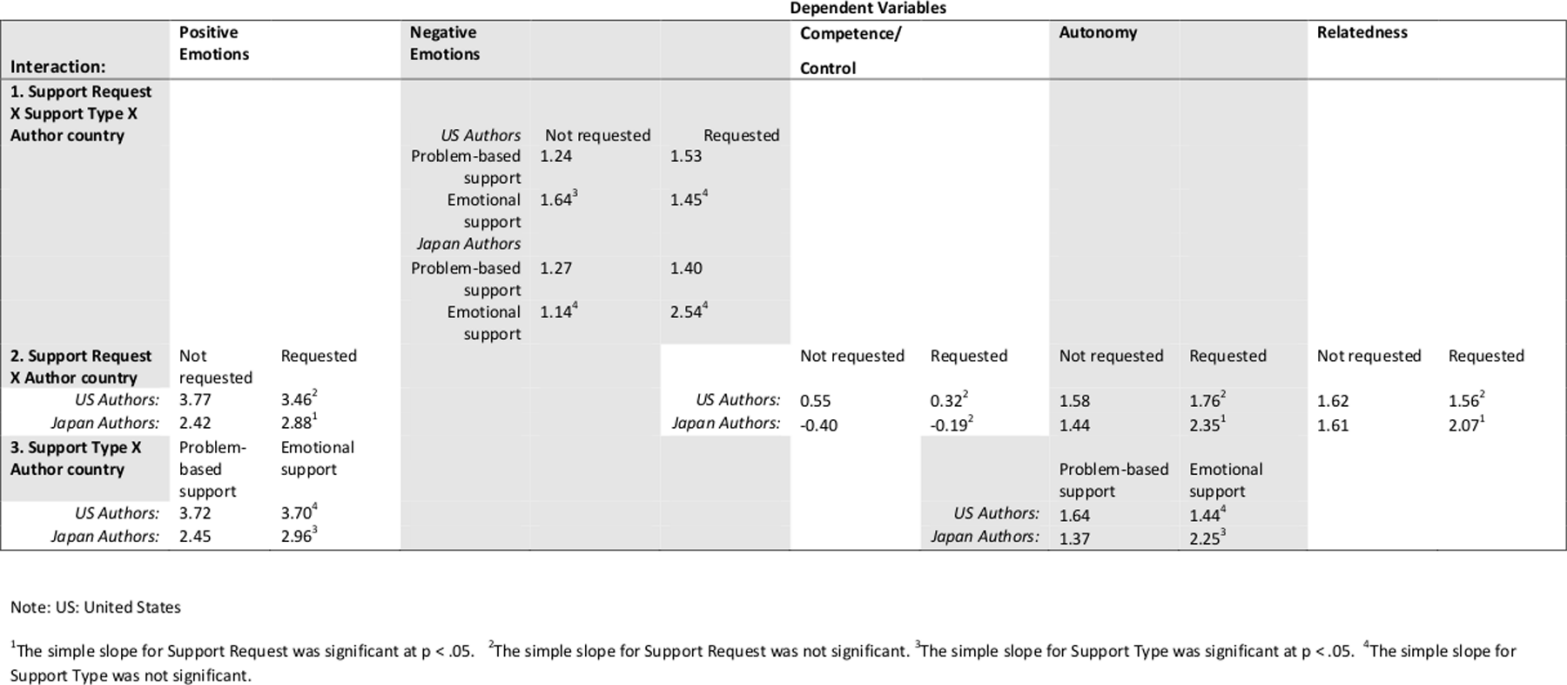
Relevant means and simple slopes results for significant coefficients, Stage 1 dependent variables (Self-rated variables).

**Fig 2:**
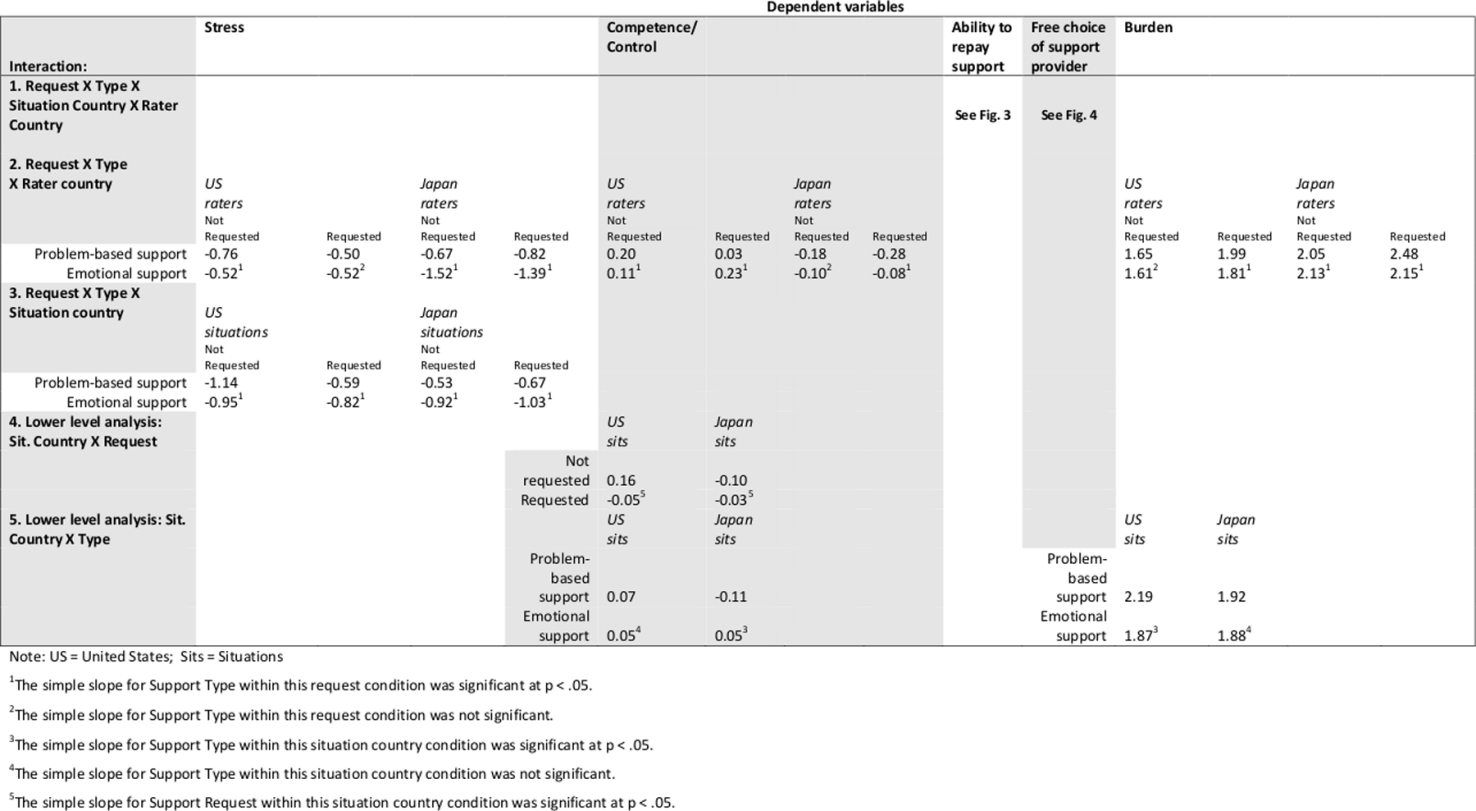
Relevant means and simple slopes results for significant coefficients, Stage 2 dependent variables.

**Fig 3:**
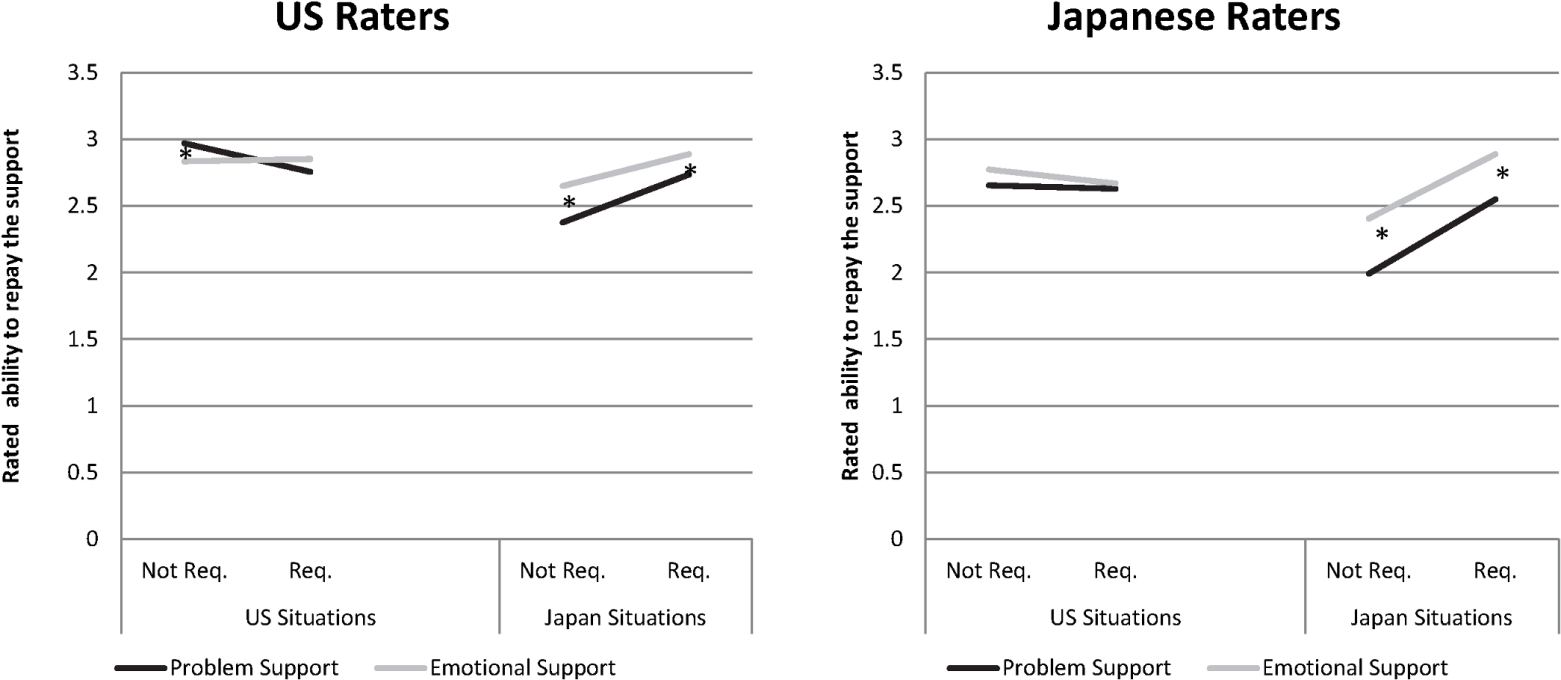
Estimated ability to repay the support-provider. Four-way interaction between Support Type, Support Request, Rater country, and Situation country. Asterisks indicate significant simple slopes comparing emotional to problem-based support. Not req = Not requested. Req. = Requested.

**Fig 4:**
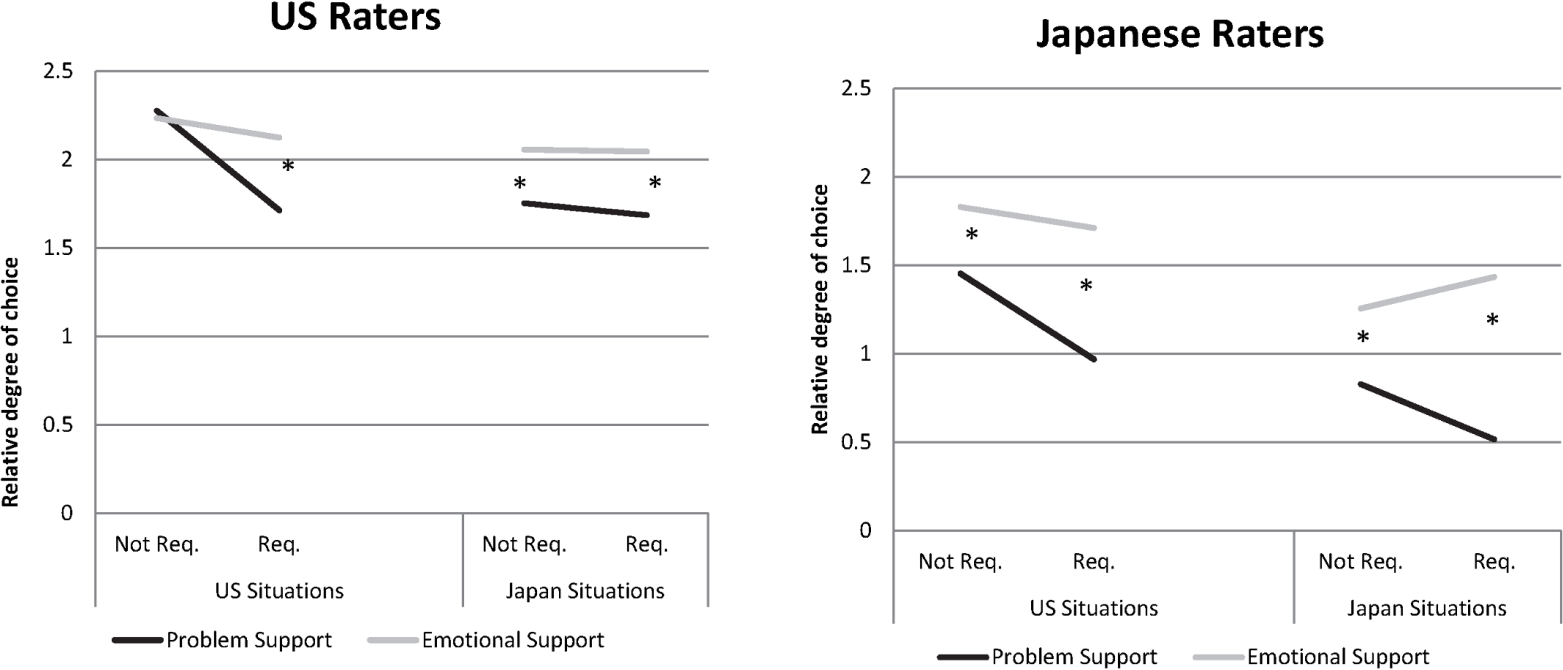
Rated free choice (vs. obligation) of the support-provider. Four-way interaction between Support Type, Support Request, Rater country, and Situation country. Asterisks indicate significant simple slopes comparing emotional to problem-based support. Not req = Not requested. Req. = Requested.

Significant results from Stage 1 analyses are in Table 3, and estimated cell means and simple slopes analyses for significant interactions are reported in Fig 1. Results from Stage 2 analyses are in Table 4, and estimated cell means and simple slopes for significant interactions are reported in Figs 2–4. Hypotheses 1–3 were tested with both Stage 1 and Stage 2 variables. Hypothesis 4–6 were tested in Stage 2 variables.

**Table 3:** Coefficients for analyses of Stage 1 dependent variables (Self-rated variables).

	Dependent Variable				
	Positive Emotions	Negative Emotions	Competence/Control	Autonomy	Relatedness
**1. Full model test:**Support Request X Support Type X Author country(γ_31_)	γ_31_ = -0.00 (.80) ns	γ_31_ = 1.79 (.63) **	γ_31_ = -1.19 (1.23) ns	γ_31_ = -0.86 (1.14) ns	γ_31_ = -0.98 (1.02) ns
**2. 2-way interactions (computed separately if full model is ns)**Support Request X Author country(γ_11_)	γ_11_ = 0.78 (.29) *		γ_11_ = 0.55 (.31) p = .07	γ_11_ = 0.73 (.41) p = .08	γ_11_ = 0.57 (.30) p = .06
3. Support Type X Author country(γ_11_)	γ_11_ = 0.68 (.35) p = .06		γ_11_ = 0.64 (.41) p = .12	γ_11_ = 1.08 (.49) *	γ_11_ = 0.24 (.37) ns

Note: For simple slopes analyses, see Fig 1.

* p < = .05

** p < .005.

For competence/control, we also observed a significant main effect for Author Country, γ_01_ = -0.87 (.24) **, such that American authors rated their competence/control higher than Japanese. US: United States; Jpn: Japan

**Table 4:** Coefficients for analyses of Stage 2 dependent variables.

	Dependent Variable				
	Stress	Competence/Control	Ability to repay support	Free choice of support provider	Burden
1. Request X Type X Situation Country X Rater Country (γ_71_)	γ_71_ = 0.35(.25) ns	γ_71_ = -0.23(.22) ns	γ_71_ = 0.36(.15) *See Fig 3	γ_71_ = 0.52(.23) *See Fig 4	γ_71_ = 0.15(.17) ns
2. Request X TypeX Rater country(γ_31_)	γ_31_ = 0.54 (.10) **	γ_31_ = - 0.17(.10) p = .08			γ_31_ = -0.27(.10) *
3. Request X Type X Situation country(γ_70_)	γ_70_ = 0.45 (.13) **	γ_70_ = -0.07(.11) ns			γ_70_ = 0.07(.08) ns
4. Lower level analysis: Sit. Country X Request (γ_40_)		γ_40_ = 0.27(.04) **			γ_40_ = -0.06(.04) ns
5. Lower level analysis: Sit. Country X Type(γ_40_)		γ_40_ = 0.18(.05)**			γ_40_ = 0.28(.04)**

Note. For simple slopes analyses, see Fig 2. US Sit. = US Situation. Japan Sit. = Japanese Situation; Prob. = Problem-based support. Emo = Emotional support; Req. = Requested support. Not Req. = Un requested support.

#### Effects of Support Request

Our first two hypotheses concerned support request and support type. In Stage 1 data, H1 was tested by Support Request X Country interactions. Although we predicted that EuA would benefit from asking for support, counter to our hypothesis, EuA actually self-reported more positive emotions and higher feelings of control (Table 3, Row 2 and Fig 1) during *unrequested* support situations. In addition, counter to predictions, Jpn authors benefited from *requested* support, reporting relatively higher feelings of competence, autonomy, and relatedness in the requested support situations (Table 3, Row 2 and Fig 1). One explanation is that in our study, Japanese could select only those situations in which they felt especially comfortable requesting support. As well, most of the support came from friends, which past work has shown to be a more comfortable target of East Asian support requests[1,6]. In addition, our design may have primed Japanese to describe some examples of *amae*, a positive social interaction in Japanese contexts.

In Stage 2 data, support request did not predict raters’ reactions to situations, at least at the main effect level (Table 4). Later we report interactions between support request and support type.

#### Effects of Support Type

H2 was tested by Support Type X Country interactions. In the Stage 1 data, as predicted, Japanese authors reported stronger positive emotions and much higher ratings of autonomy in emotional support situations compared to problem-support situations (see Table 3, Row 3, and Fig 1). In contrast, EuAs rated their own problem-based and emotional support about equally.

H2 was supported in Stage 2 data as well (Table 4 and Fig 2). First, as predicted, for both Japanese raters and Japanese situations, emotional support situations were lower in rated *stress* than problem-based support. And for both Japanese raters and Japanese situations, rated *competence* was relatively higher for emotional support situations. Japanese emotional support situations were rated significantly higher in *ability to repay* the support than Japanese problem-support situations (and especially so by Japanese raters; see Fig 3, right panel). These three results from the Stage 2 data suggest that emotional support’s benefit is carried in Japanese situations, not only in Japanese raters.

#### Support Type X Support Request interactions

In an exploratory mode, we tested the interaction of Country, Support Type, and Support Request. In the Stage 1 data, one such three-way interaction was significant, for negative emotions, (Table 3, Row 1) such that EuAs rated their own unrequested, problem-based support situations especially low in negative emotions.

In the Stage 2 data, we explored the same three-way interactions and found the same pattern favoring EuA unrequested, problem-based support (see Table 4 and Fig 2). For both EuA raters (Table 4, Row 2) and EuA situations (Table 4, Row 3) the combination of unrequested and problem-based support was significantly lowest in *stress*. EuA raters also felt more *competent* in support that had been unrequested and problem-based (Table 4, Row 2). And EuA unrequested, problem-based support, was rated as highest in *ability to repay* (but only by Americans; Fig 3). Finally, emotional support situations were rated significantly higher in free choice for all situations with the exception of (again) EuA, unrequested support, which EuA rated problem-based and emotional support as equally freely chosen.

Overall, it seems that unrequested, problem-based support was rated most positively by EuA or in EuA situations. Thus support type and support request do appear to interact, at least in the situations generated by or rated by Americans.

#### Needed support in Japan

H3 predicted that support would be especially effective in Japan if it was needed. Indeed, although we found, surprisingly, that Jpn authors preferred requested support in our study, we reasoned that the positive effect of asking for support may have been attributable to how much this requested support was needed. Therefore, we followed up the unexpected result for requested support in Japan with additional analyses to see if perceived need for support was a mediator of the observed pattern. We first tested the Stage 1 model using self-reported “need for help” as a dependent variable and found that while all participants needed support more when they asked for it, the relation between asking and needing for Japanese authors was especially strong (US slope: b = 0.49(.16) p < .001; Japan slope: b = 1.32 (.17) p < .001; Country X Request interaction: γ_11_ = 0.82 (0.25) *p* < .001). Next, we found that differences in degree of need mediated the Support Request X Author Culture interactions, at least for the DV’s of autonomy and relatedness. Following guidelines from Muller, Judd, and Yzerbyt[47] on mediated moderation, we found that the observed Country X Request effect on *autonomy* reduced in size from γ_11_ = 0.84 (0.40) *p* = .04 to γ_21_ = 0.31 (0.35) *p* = .38 (Sobel = 2.69 (.16), p < .01), and the Country X Request effect on *relatedness* reduced in size from γ_11_ = 0.64 (0.30) *p* = .03 to γ_21_ = 0.44 (0.29) *p* = .13 (Sobel = 2.55 (.16), p = .01), when the Country X Need effect was added to the equation. (As required in HLM, the continuous mediator, Need, was also in the equation, and to be consistent with other analyses, the Country X Situation Type interactions remained in the equations.) The Country X Request effect on *control* did not significantly decrease (γ_11_ = 0.56 (0.30) *p* = .07 to γ_21_ = 0.65 (0.35) *p* = .06, Sobel = 1.37 (.08), p = .17).

The foregoing analyses were not planned originally and were only conducted in light of the unexpected result that Japanese reported more positive reactions to requested support. Nevertheless, the results of these analyses support H3, and suggest that Japanese felt more autonomy and relatedness from requested support because they actually needed support more (compared to European-Americans), and because needing support was more strongly associated with autonomy and relatedness for Japanese (compared to European-Americans).

Further tests of H3 are discussed in a later section.

#### Rated choice of the provider

H4 predicted that EuA raters would perceive more free choice on the part of the person providing the support, and that EuA situations would also convey more free choice compared to Jpn raters and situations. The highest-level, 4-way interaction was significant (see Fig 4). We observed that, consistent with H4, American raters perceived more choice by support-givers overall than Japanese raters did. More importantly, both American and Japanese raters perceived more choice by support-givers in American situations than in Japanese situations.

#### Competence/control

H5 predicted that EuA raters would report feeling more competent or efficacious in all situations, and that EuA situations would also convey more control and competence, compared to Jpn raters and situations. The Stage 1 predicted means (Table 3, Fig 1) for competence showed that for all situation types, Americans rated their competence at or above the midpoint, whereas Japanese rated their competence below the midpoint, a significant main effect. In Stage 2, EuA support situations were rated as higher in competence than Jpn problem-based support (Table 4, Fig 2); however, note that Jpn emotional support was not significantly different in evoked competence from EuA situations (as predicted by H2).

#### Burden

H6 predicted that Jpn raters and Jpn situations would rate or be rated higher in perceived burden to the support-provider, compared to EuA raters and situations. Japanese raters rated their perceived burden to be significantly higher overall than American raters (Table 4, Row 2; Fig 2). However, our results show that these cultural differences in burden are in the eye of the beholder—not communicated by the situation: Although Japanese *raters* felt higher levels of burden, Japanese *situations* were not higher on this variable (Table 4, Row 5; Fig 2).

In addition, one might predict burden ratings to be higher for requested support and problem-based support; this pattern was partly supported (Fig 2) but did not interact with culture.

#### Summary and discussion

Results did not support H1. Surprisingly, EuAs rated their own social support more positively if they did not ask for it. However, interactions of support request and support type suggest that it was mainly unrequested *problem-based* support that was rated especially favorably. Such help stood out as low in stress, high in competence, more repayable, and freely offered when rated by EuAs, within EuA situations, or both. Past work [20] has shown that social support is more prevalent in EuA cultural contexts. In these everyday contexts, such freely-offered problem-based support may feel good because it’s simply a common or everyday part of American interactions.

H1 was also unsupported because Jpn rated their own social support more positively if they had asked for it. However, our follow up analyses suggested that Japanese (compared to EuA’s) asked for support more in situations that they rated higher in need, and needing support was more strongly associated with autonomy and relatedness in Japanese contexts. Therefore, in the present study, Japanese benefited more from requested support because they needed such support relatively more and, in addition, they benefit relatively more from needed support (compared to EuA’s). Future research should replicate this post-hoc mediated moderation pattern, in addition to testing the temporal sequence of events.

Results supported the idea that Japanese situations and raters favor emotional support (H2). Japanese raters and situations felt lower in stress, more competent, more repayable, and more freely offered when they were emotional. The flipside is that for Japanese contexts and/or raters, problem-based support situations feel more stressful, less competent, and so on. It seems possible that problem-based support accompanies a sense of officiousness and social obligation. The officiousness may contrast with the preferred, socially authentic empathy of emotional support.

EuA raters and EuA situations did indicate, as expected, that providers acted out of free choice and that support made recipients feel competent (H4 and H5). And Jpn raters, supporting H6, reported worrying about burdening the support provider, but the perception of burden was not conveyed by Japanese situations—it was only in the eye of the beholder.

### Further Analyses Testing H3

To further test Hypothesis 3, we ran a set of analyses to test the degree to which the author’s self-reported need, compared to other variables, predicted positive situation outcomes. If support in Japan is most effective when it is responsive, then *needed* support should matter the most in Japanese situations.

We separated the situations into four subsets: American problem-based support, Japanese problem-based support, American emotional support, and Japanese emotional support (see Table 5). As the dependent variable, we used coders’ ratings of the positivity of the ending of the situation. Coded positivity was the only variable that explicitly separated the positivity of the *ending* of the situation from the severity of it at the start.

**Table 5:** Stage 2 predictors (standard errors) of coded positivity at the end of the situation, in problem-based and emotional support situations.

Situation Type	US Problem Support Situations	Japan Problem Support Situations	US Emotional Support Situations	Japan Emotional Support Situations
1. Intercept	0.73(.06)**	-0.50(.04)**	1.69 (.05)**	-1.91(.36)**
2. Support Request (-1 = no, 1 = yes)	0.03(.01)*	-0.10(.01)**	0.02(.02)ns	0.12(.02)**
3.Coded severity at start	0.10(.01)**	0.16(.02)**	-0.03(.01)**	0.28(.02)**
4. Author-rated need for support	0.10(.01)**	0.30(.01)**	-0.05(.01)**	0.62(.07)**
5. Stage 2 rated stress in situation	-.12(.01)**	-0.14(.01)**	-0.08(.01)**	-0.06 (.01)**
5.Stage 2-rated feelings of burden	0.08(.01)**	0.02(.01) *	0.06(.01)**	0.07(.01)**
6. Stage 2-rated chance to repay support	0.04(.01)**	0.09(.01)**	0.00(.01)ns	-0.02(.01)**
7.Stage 2 rated feelings of control/competence	0.08(.01)**	0.04(.01)**	0.06(.01)**	0.06(.01)**
8. Stage 2 provider’s degree of free choice	0.03(.01)**	0.04(.01)**	0.06(.01)**	-0.00(.01) ns

Notes. Author-rated predictors were collected at Stage 1. Other-rated predictors were collected at Stage 2. Coded positivity (the DV) and coded severity at start were rated by trained coders.

* = *p* < .05

** = *p* < .005.

Predictor variables (see Table 5) included the main effect predictor Support Request (effect coded to aid interpretation of other predictors), coded situation severity (as a control variable), Stage 1 self-rated need for the support as the target predictor, and Stage 2 ratings. Because of the proposed Japanese model of support as empathic assurance, we predicted that self-rated *need* for the support should be more highly correlated with situation outcome for Japanese situations than American ones. Because of American models of help as autonomous, we included *competence* and the *free choice* of the provider as possible correlates of positive outcomes for American situations.

The tested model was as follows:

DV (coded positive outcome) = β_0j_ + β_1j_ (situation request) + β_2j_ (coded severity of the situation) + β_3j_ (Stage 1 need for support) + β_4j_ (Stage 2 burden) + β_5j_ (Stage 2 repay rating) + β_6j_ (Stage 2 competence) + β_7j_ (Stage 2 provider’s free choice) + β_8j_ (Stage 2 provider’s stress) r_ij._

β_0j_ = γ_00_+ u_0j_

β_1j_ = γ_10_ + u_1j_

β_2j_ = γ_20_ + u_2j_

β_4j_ = γ_40_ + u_4j_

β_5j_ = γ_50_ + u_5j_

β_6j_ = γ_60_ + u_6j_

β_7j_ = γ_70_ + u_7j_

#### Results and discussion

Coefficients are displayed in Table 5. Notably, intercepts for Japanese situations were lower than those for US situations. Either the positivity of outcomes is truly lower in Japan, or the Japanese coders simply tended to use the more negative end of the coding scale; either interpretation is consistent with past work showing lower levels of positivity in Japanese contexts [48]

Second, as found in Stage 1 results, requested support was more positive in all Japanese situations.

Third, in situations of both types, self-rated degree of needing the support was strongly related to how positive the outcome was. However, the slopes were much stronger in Japanese than US situations (supporting H3). Relatedly, in Japanese situations, situation severity at the start of the situation was more strongly associated with positive outcomes, compared to US situations. These results are consistent with the hypothesis that social support is experienced more positively in Japan when it is sensitive to one’s needs and to the severity of the situation.

The results did not show that competence and free choice were especially strongly correlated with positive outcomes in US situations. Although American problem support is experienced positively, American support is not *uniquely* more positive when it feels competent, repayable, freely chosen, and so on. Thus, the main result of these analyses is that the outcome of Japanese situations is very strongly linked to how much support was needed.

## General Discussion

Our study collected hundreds of examples of received social support from Japanese and middle-class, European American (EuA) samples. By interpreting multiple ratings from different perspectives, we are able to sketch a description of the everyday social support in these two cultures. We classify this study as a quantitative step towards “thick description”[49] of two cultural worlds. Table 6 summarizes the support for all hypotheses.

**Table 6:** Summary of Research Hypotheses.

	Prediction forAmericans	Prediction forJapanese	Study Outcome
H1 Support request	Support more effective when requested	Support less effective when requested	Not supported in Stage 1 data:Americans found unrequested support more supportive (especially unrequested, problem based support) Japanese found requested support more supportive (because requested support was relatively more needed in Jpn and needed support is especially more positive in Jpn).Not supported in Stage 2 data;Instead, American unrequested, problem- based support more effective
H2 Support type		Emotional support will be more effective than problem based support	Supported in Stage 1 dataSupported in Stage 2 data
H3 Role of support need		Needed support will be more effective	Supported in Stage 1 data; needed support was more effective, especially in JapanSupported in Stage 2 data; need is strong predictor of Japan situations’ outcomes
H4 Free choice and repayment (autonomous transactions)	Free choice and repayment higher in US situations and by US raters		Not tested in Stage 1 dataSupported in Stage 2 data
H5 Competence/control	Feelings of control and efficacy higher in US situations and by US raters		Supported in Stage 1 dataSupported in Stage 2 data (problem support only)
H6 Concern about burdening the support provider		Concern about burdening the support provider higher in Jpn situations and by Jpn raters	Not tested in Stage 1 dataSupported in Stage 2 data (raters only)

### Summary of Findings

#### Everyday support transactions in middle-class European-American contexts

Our results updated the picture of social support in the United States. Rather than simply as a response to stress, our data suggest that EuA social support is practical, frequent, easily exchanged, and helpful. It seems that EuA experience problem-based support as a natural part of everyday interactions, and even unrequested advice or assistance is experienced positively.

Although receiving unrequested help could threaten one’s competence, the authors of EuA situations seem to have emphasized support givers’ own free will and the recipient’s ability to repay it—US situations were rated higher in the ability to repay support than Japanese situations, and EuA raters endorsed this dimension more than Japanese did. People’s ability to repay support (Fig 3) taps into the equitable reciprocation of support that seems important to North Americans [24,26]. Support was also freely given—American *raters* judged all support-providers as higher in free choice than Japanese raters did, and American *situations* were judged to be higher on this dimension, too. Rather than reporting a threat to one’s competence, the authors of unrequested problem support situations seem to have emphasized the competence they felt in these situations.

#### Officious support in middle-class Japanese contexts

In Japan, we found that problem-based support may be much more common than emotional support, but it seems overall to be less helpful. Apparently, when problem-based advice or assistance is exchanged in Japan, recipients react negatively. Japanese raters felt less calm and less competent when imagining receiving problem-based support. Japanese situations communicated this negative feeling too—problem based support situations in Japan were rated by all as feeling less calm, less competent, and less freely offered. This phenomenon, which we tentatively label “officiousness” matches observations by anthropologist Kondo[50]. Problem-based support in Japan may be interpreted not as a freely offered boost to competence, but as irritating—at least if it is offered to someone who doesn’t need it[38].

#### Empathic assurance in Japan

In complement to the negative officiousness of problem based support, our results suggest positive outcomes of emotional support in middle-class Japanese contexts. Although the social support our Japanese participants recalled was relatively unlikely to be emotional in nature, emotional support is experienced more positively. Our data strongly supported a model of support in which empathy and responsiveness drives support. The emotional support provided by Japanese seems to be especially effective in evoking calmness and competence (see Fig 2), regardless of culture. Emotional support was perceived as freely given, even when it was requested (see Fig 4). And even when it is requested, emotional support put less burden on the provider than problem-based support did (see Fig 3). Importantly, the more the support is needed—whether it is problem-based or emotional—the better the outcome (Table 5). Apparently sincere and responsive support is most effective for Japanese. Although we found, in contrast to past work, that Japanese *requested* support was generally more positive, our present methods were more naturalistic, so people may have described requested support from close friends—perhaps even *amae*, an emotional script in which a person behaves inappropriately and a close other indulges him or her[29,51,52].

Emotional support, as operationalized in the present study, overlaps conceptually with past research that has studied perceived emotional support[8], implicit support[14],emotional support[20], social assurance [35], and *amae* [52]. Our data provide a meaningful replication to past research—in East Asian contexts, emotional support is described and experienced positively.

#### Other notable replications

Using a different method from past research, we replicated Kim et al.’s[6,53] results that East Asians report greater feelings of burden toward support-providers. Japanese raters reported especially high feelings of burden for requested, problem-based support (see Fig 2). However, burden reflects attitudes of the raters, not qualities of the situations, because Japanese *situations* were not rated by Stage 2 raters as being any higher in perceived burden (H6).

In a second important replication (H5), the present study found that EuA situations were rated higher on control (efficacy, competence) than Japanese situations, and also that EuA reported higher overall competence than Japanese did, replicating past work[10]. This result suggests that even in social support situations (a different kind of situation than was collected before), EuA situations evoke feelings of control.

#### Support type and support request interactions

One strength of the present research was that we treated support request and support type as separate dimensions. Our results suggest that support type and support request may interact. When people receive emotional support, it seems to be experienced positively whether it is requested or not. Intuitively, it might seem awkward to ask for emotional support (e.g., “Will you help me feel better?”), and indeed these situations were rare (see Tables 1 and 2). People asked for emotional support mostly from parents and close friends. We may conclude that, at least when people provide their own naturalistic examples, emotional support helps whether it is requested or not.

### Contributions and Limitations

The present research studied social support to include everyday—not only stressful—situations cross-culturally. We attempted to capitalize on the strengths of situation sampling, which provides a peek into the daily lives of people in different cultures and measures the extent to which those lives can evoke reactions in others. We tested how different cultural models of social support outlined by other researchers[6,8,20] might be portrayed in people’s descriptions of their received social support. Our data are naturalistic and correlational. We coded and analyzed actual situations, retaining the original descriptive language that our authors used. At all stages of situation sampling, cultural norms, discourse conventions, and personal goals can potentially distort what gets described. Rather than detracting from the interpretation of the data, we attempted to quantify the impact of these norms and conventions through our analyses of people’s ratings of the situations.

There are a number of avenues for follow up. Daily diary studies could replicate the finding that for middle-class Americans, offers of practical support are ubiquitous and positive, whereas in Japan, they feel a bit officious. Such work could isolate factors that make everyday American support more positive than Japanese support. Future work is important to make psychology’s picture of social support even more culturally and contextually valid.

## Supporting Information

S1 Datasets Compressed file contains four SPSS .sav datasets: S1: Stage 2 Level 1 Data situation as unit PLOS one (Stage 2 data, situation as unit of analysis, others rating situations); S2: Dataset Stage 2 level 2 data respondent as unit PLOS one (Stage 2 data, situation as unit of analysis, others rating situations); S3: Stage 1 level 1 Data situation as unit PLOS one (Stage 1 data, person as unit of analysis, self-reporting on situations); and S4: Stage 1 level 2 data respondent as unit PLOS one(Stage 1 data, person as unit of analysis, self-reporting on situations)
(ZIP)
